# Health problems account for a small part of the association between socioeconomic status and disability pension award. Results from the Hordaland Health Study

**DOI:** 10.1186/1471-2458-11-12

**Published:** 2011-01-06

**Authors:** Kristian Amundsen Østby, Ragnhild E Ørstavik, Ann Kristin Knudsen, Ted Reichborn-Kjennerud, Arnstein Mykletun

**Affiliations:** 1 Department of Mental Health, Norwegian Institute of Public Health, Oslo, Norway; 2 Department of Health Promotion and Development, Faculty of Psychology, University of Bergen, Bergen, Norway; 3 Department of Mental Health, Norwegian Institute of Public Health, Oslo, Norway; Institute of psychiatry, University of Oslo, Oslo, Norway; 4 Department of Epidemiology, Columbia University, New York, USA; 5 Department of Mental Health, Norwegian Institute of Public Health, Oslo, Norway; Faculty of Psychology, University of Bergen, Bergen, Norway

## Abstract

**Background:**

Low socioeconomic status is a known risk factor for disability pension, and is also associated with health problems. To what degree health problems can explain the increased risk of disability pension award associated with low socioeconomic status is not known.

**Methods:**

Information on 15,067 participants in the Hordaland Health Study was linked to a comprehensive national registry on disability pension awards. Level of education was used as a proxy for socioeconomic status. Logistic regression analyses were employed to examine the association between socioeconomic status and rates of disability pension award, before and after adjusting for a wide range of somatic and mental health factors. The proportion of the difference in disability pension between socioeconomic groups explained by health was then calculated.

**Results:**

Unadjusted odds ratios for disability pension was 4.60 (95% CI: 3.34-6.33) for the group with elementary school only (9 years of education) and 2.03 (95% CI 1.49-2.77) for the group with high school (12 years of education) when compared to the group with higher education (more than 12 years). When adjusting for somatic and mental health, odds ratios were reduced to 3.87 (2.73-5.47) and 1.81 (1.31-2.52). This corresponds to health explaining only a marginal proportion of the increased level of disability pension in the groups with lower socioeconomic status.

**Conclusion:**

There is a socioeconomic gradient in disability pension similar to the well known socioeconomic gradient in health. However, health accounts for little of the socioeconomic gradient in disability pension. Future studies of socioeconomic gradients in disability pension should focus on explanatory factors beyond health.

## Background

The high proportion of the population on disability pension is a major concern in many developed countries. In most OECD countries (Organisation for Economic Co-operation and Development) the rate of inhabitants on disability pension is on a slight but steady rise [1,2]. In Norway the costs of disability pension was estimated to a total of 52 billions NOK in 2007 [3], accounting for approximately 2.3% of the country's Gross Domestic Product.

In many countries, including Norway, medical conditions are the only formally accepted causes for being granted a disability pension. The Norwegian National Insurance Act states, in line with most OECD countries, that to be considered for a disability pension your earning capacity must be reduced due to permanent illness, injury or impairment. Nevertheless, both clinical experience and results from previous studies indicate that medical conditions are not the only decisive factors in these matters, and the labour marked situation (level of education, employment status and occupational factors) are also of considerable importance [4-8]. For instance, in a report from 2010, Bratsberg et al shows that a large fraction of disability insurance claims can be attributed to reduced employment opportunities [5].

Several studies have shown that low socioeconomic status (SES) increases the risk of disability pension [6,8-10]. A Danish study found a 3-4-fold increased risk of disability pension among persons with seven years of schooling only, compared to persons with a university degree, after adjusting for social and demographic factors [9]. In a Norwegian 10-year longitudinal study of a population aged 20-50 years at baseline, men and women with elementary school had adjusted relative risks of 2.9 and 4.8 respectively for being granted a disability pension when compared to those with higher levels of education [7].

Differences in health between different socioeconomic strata are expected to account for some of the differences seen in rates of disability pension, as low SES is associated with both worse health [4,10], higher mortality [10,11], less favourable health behaviour [10,12], and an increased risk of sickness leave [13-15]. But to the best of our knowledge, no previous study has directly examined how much of the increased risk of disability pension associated with low SES that is actually explained by health.

In Norway, large population based health surveys can be linked to complete national registries of awarded disability pensions by personal identification numbers. They are thus well suited for the purpose of studying the associations between SES, health and disability pension as it is possible to adjust for a wide range of health factors of both somatic and mental nature.

The aim of our study was to estimate the proportion of the increased risk of disability pension associated with lower SES that can be explained by actual differences in health.

## Methods

### The Hordaland Health Study

The data collection was conducted as part of the Hordaland Health Study (HUSK) in collaboration with the Norwegian National Health Screening Service and the local health services. HUSK is one of several Norwegian population based health surveys, and took place between 1997 and 1999 in Hordaland county, a county in western Norway with both urban and rural areas.

**Figure 1:**
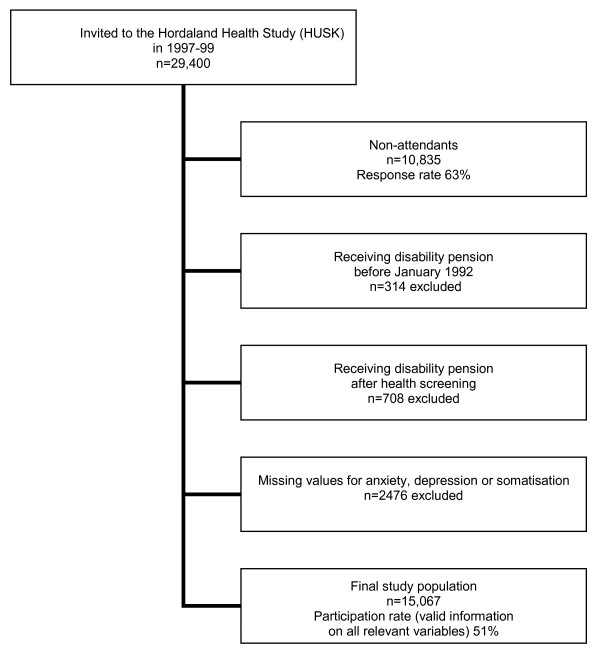
**Study group**.

All inhabitants in Hordaland County born between 1953 and 1957 were invited to participate (Figure 1), giving a target population of 29,400 subjects (15,051 men and 14,349 women), aged 39-46 years at the time of the study. The participants responded to questionnaires on various health related matters and sociodemographic factors, including the highest completed level of education. Participants also went through a clinical examination, with measures on height, weight, blood pressure and blood level cholesterol. 18,565 persons (8585 men and 9980 women) met to the clinical examination for inclusion in the study, giving a response rate of 63% (57% for men, 70% for women).

### Exposure: Socioeconomic status

Several measures of SES have been used in previous research, including education, income (personal or household) or classification of occupation. Education has been shown to be a particularly good measure of SES in health related issues [16,17]. In addition, level of income is generally reduced when on disability pension, and this would diminish the relevance of income as a measure of SES with our approach.

As a measurement of SES we used self-reported information on achieved level of education, stratified into three groups; compulsory school only (9 years of schooling), high school (a total of 12 years of schooling), and higher education (more than 12 years of schooling, corresponding to a college or university degree).

### Outcome: Disability pension

According to the law, a permanent disability pension is awarded for life for chronic conditions after proper treatment and rehabilitation is performed. Consequently, we wanted to compare current recipients of permanent disability pensions to those who do not receive such a pension (whereof the majority are in labour work).

Using personal identification numbers, which are issued to all Norwegians by the state at time of birth (for immigrants at time of immigration) the health survey was linked to the National Insurance Administration's (NIA) records on disability pensioning covering the period January 1992 to December 2004. These registries are updated annually, and their accuracy is well documented [13]. We excluded, however, all subjects who were granted a disability pension after the collection of health data in HUSK (N = 708), as these disability pensions could be based on health problems originating after the assessment of health data in HUSK.

NIA has recorded exact dates for initiation of all disability pensions granted after January 1992, but for disability pensions granted before this, we did not have precise data on time of initiation. A disability pension granted early in life may influence on the education level reached. Thus all subjects who had been granted a disability pension before January 1992 (N = 314, aged 32-40 years at the time) were also excluded from the analyses (Figure 1).

For participants included in the study, the time range assessing disability pension was therefore from 1992 until their time of participation in HUSK (which for each participant was some time in the period of 1997-1999).

### Health variables

The Norwegian National Insurance Act states, in line with most OECD countries, that to be considered for a disability pension your earning capacity must be reduced due to permanent illness, injury or impairment. Thus we adjusted for as many such health factors as possible (both somatic and mental) to estimate how much of the relationship between SES and disability pension was left unexplained.

Mental health problems have been shown to be considerable risk factors for disability pension, including disability pensions awarded for somatic disorders [18,19]. Symptoms of anxiety and depression were assessed in HUSK with the Hospital Anxiety and Depression Scale (HADS) [20]. The scale consists of 14 items, 7 related to anxiety (HADS-A) and 7 related to depression (HADS-D). The two HADS sub-scales are often used with a cut-off value for probable positive cases [21], but have been shown to be better predictors of impairment when used dimensionally rather than categorically [20]. Thus, in this study, the HADS sub-scales were used as two separate ordinal variables.

As a measure of somatic conditions the subjects were asked whether they "have or have had" coronary infarction, angina, stroke, diabetes, asthma or multiple sclerosis. The number of conditions checked was summed up in one ordinal variable; *number of self-reported somatic conditions*. Scores above 3 was collapsed at 3. The variable thus included 4 categories (0-3). The participants were asked if they had taken any medications the previous day, and, if so, for which medical condition and the name of the medication. On the basis of this information, a team of physicians appointed appropriate diagnoses based on the International Classification of Primary Care system (ICPC). An ordinal variable describing the number of different diagnoses for which the respondent received medications was established; *number of medicated diagnoses *with values ranging from 0 to 3 (scores above 3 collapsed).

As a substantial proportion of disability pensions are likely to be granted on basis of more diffuse pain disorders [22], we also adjusted for somatic symptoms. Participants were asked about 17 somatic symptoms from the ICD-10 research criteria for Somatisation disorder (F45.0). For each symptom, all participants checked off how often they experienced the symptom: "almost never", "rarely", "sometimes", "often" or "almost always". Responses were coded from 0 to 4 and summed up in one continuous variable [23].

Musculoskeletal pain and disorders rank among the most frequent causes of disability pensioning [24], and fibromyalgia has been one of the most frequent single diagnosis causing disability pensions in Norway [25]. In our study the participants were asked if they "suffered from Fibromyalgia/Fibrositis/Chronic pain syndrome", and the answers were entered into a dichotomous variable (yes/no). The participants were also asked if they during the previous year had been suffering from pain and/or stiffness that lasted for at least 3 consecutive months, and if so, they were asked to specify the localization of this pain to any of the following 10 anatomical locations: neck, shoulders, elbows, hands/wrists, chest/abdomen, upper back, lower back, hips, knees and ankles/feet. One dichotomous variable was established for each pain site.

Obesity is a known risk factor for sickness absence and disability pension [26,27], and weight and height were measured at the clinical examinations in HUSK. A Body Mass Index (BMI) of 30 or more was classified as "obesity" in the analyses and treated as a dichotomous variable.

### Missing values

A total of 2476 participants were excluded from the study for having missing information on symptoms of anxiety, depression or somatisation (Figure 1) [28]. Missing data on self reported somatic diagnoses (n = 44), diagnoses based on medication (n = 267) and on fibromyalgia (n = 56) were substituted by the mode [29]. Missing data for measured BMI (n = 27) was substituted by mean value of 25.4 [29].

### Analyses

Univariate logistic regression analysis was applied to estimate the unadjusted Odds Ratios (ORs) for being granted a disability pension with level of education as independent variable and higher education (>12 years) as the referent group. Multivariate analyses were then performed controlling for all relevant health variables measured in the survey, modelled as continuous or dichotomous variables as described above. All analyses were performed in SPSS version 14.0 for Windows.

The proportion of the association between educational level and disability pension that was accounted for by health was calculated as

$$(\beta_{\text{unadjusted}}-\beta_{\text{adjusted}})/\beta_{\text{unadjusted}}$$

where β is the regression coefficient (whereof OR is calculated as e^β^).

Stratified analyses for men and women were performed to explore eventual sex differences.

To analyse associations between health and socioeconomic status and between health and disability pension, we used one-way ANOVA tests for the continuous variables and chi-square tests for the dichotomous variables. For these particular analyses of association, the ten dichotomous variables on localized pain/stiffness were summed up in one variable indicating number of pain sites, and analysed as one continuous variable.

### Ethics

The study protocol was approved by the Regional Committee for Medical Research Ethics, Western Norway and by the Norwegian Data Inspectorate. All HUSK participants gave their written informed consent when they met at the examination premises.

## Results

The final study sample consisted of 15,067 subjects, 51% of those invited to HUSK (Figure 1). Among the participants, 17% had compulsory school as their highest level of education, 46% had high school, and 37% higher education (university or college). Of the total sample 2.1% had been granted a disability pension (Table 1).

**Table 1:** Population characteristics

	%	Mean (SD)
**Age^1^**		42.6 (1.50)
**Sex (male)**	45.9	
**Disabiliy pension**	2.1	
**Educational level (years of schooling)**		
**Compulsory school (9 years)**	17.3	
**High school (12 years)**	45.9	
**Higher education (> 12 years)**	36.8	
		
**Average symptom score anxiety (HADS-A)**		4.60 (3.26)
**Average symptom score depression (HADS-D)**		3.19 (2.89)
**Number of somatic diagnosis**		0.08 (0.29)
**Number of pharmacological treatments**		0.08 (0.33)
**Somatic symptoms**		11.27 (7.88)
**Body Mass Index (kg/m^2^)**		25.31 (3.74)
**Obesity (BMI ≥ 30)**	10.6	
**Self-reported fibromyalgia**	5.6	
**Have had lasting pain/stiffness previous year**	42.0	
**Neck**	23.4	
**Shoulders**	25.8	
**Elbows**	7.4	
**Hands/wrists**	10.1	
**Chest/abdomen**	4.9	
**Upper back**	12.5	
**Lower back**	20.8	
**Hips**	11.6	
**Knees**	11.0	
**Feet/ankles**	8.6	

^1 ^age at participation in the health screening

In bivariate analyses, all examined health problems were increased in those receiving DP (all p < 0.01). All examined health problems were also associated with educational level in directions as expected (all p < 0.02), the exceptions being the two variables on number of somatic diagnoses and number of medicated diagnoses, whose associations were not statistically significant.

### The mediating effect of health

The unadjusted analyses indicate an increased OR for disability pension in the lowest SES groups: OR = 4.60 (95% CI: 3.34-6.33) for those with elementary school and 2.03 (1.49-2.77) for those with high school, when compared to those with higher education. Adjusting for all health variables reduced the ORs to 3.87 (2.73-5.47) and 1.81 (1.31-2.52) respectively (Table 2). Similar patterns were found when analyses were stratified for sex (Table 2).

**Table 2:** Unadjusted and adjusted odds ratios for disability pension

	Odds ratio Crude	95% CI	Odds ratio Adjusted^1^	95% CI	Proportion accounted for by health^2^
**ALL **(n = 15067)					

**Higher education (> 12 years)**	**1**		**1**		

**High school (12 years)**	**2.03**	1.49 - 2.77	**1.81**	1.31 - 2.52	**16%**

**Compulsory school (9 years)**	**4.60**	3.34 - 6.33	**3.87**	2.73 - 5.47	**11%**

					

**MEN **(n = 6909)					

**Higher education (> 12 years)**	**1**		**1**		

**High school (12 years)**	**2.34**	1.36 - 4.01	**2.00**	1.13 - 3.55	**18%**

**Compulsory school (9 years)**	**5.46**	3.10 - 9.61	**4.39**	2.39 - 8.06	**13%**

					

**WOMEN **(n = 8158)					

**Higher education (> 12 years)**	**1**		**1**		

**High school (12 years)**	**1.88**	1.29 - 2.75	**1.79**	1.19 - 2.70	**8%**

**Compulsory school (9 years)**	**4.03**	2.74 - 5.94	**3.80**	2.47 - 5.85	**4%**

**^1^Health factors adjusted for: ***Anxiety symptom score (HADS-A), Depression symptom score (HADS-D), Number of somatic diagnosis, Number of pharmacological treatments, Somatic symptoms, Obesity (BMI ≥ 30), Self-reported fibromyalgia, Lasting pain/stiffness during previous year in each of Neck, Shoulders, Elbows, Hands/wrists, Chest/abdomen, Upper back, Lower back, Hips, Knees and Feet/ankles*.

^2^***Calculated using the formula**: 1 - (ln(OR_adjusted_)/ln(OR_crude_))*

Adjustment for health reduced the effect size of the association between socioeconomic status and disability pension by 16% comparing 12 with more than 12 years of education, and by 11% comparing 9 with more than 12 years of education (Table 2). The corresponding numbers for men was 18% and 13%, compared to 8% and 4% in women. Thus, health accounted for less of the association between educational level and disability pension in women than in men.

## Discussion

Among adults in their forties, low socio-economic status, defined as a low level of education, had an independent association with disability pension, which to a limited degree could be explained by socio-economic gradients in health. Health accounted for only a limited proportion of the observed association between disability pensions and educational level. This indicates that the increased level of disability pension in those with low education can only partly be explained with socioeconomic gradients in health. Our finding calls upon explanatory factors beyond health in describing the social gradient in disability pensioning.

Even though health problems are the only formally accepted reason, a likely explanation of our results is that social or personal factors play an important role in determining who is granted a disability pension [30]. This is in line with theories promoted by other researchers; some pointing out that social or personal factors may be crucial [7,24,30,31].

A low level of education means less flexibility and fewer options when it comes to choosing profession or work place. This might increase the risk of disability pension, as there will be more obstacles to find alternative work if the current work situation is difficult. High education might likewise function as a protective factor against disability pension, as it may provide more alternatives when the work situation is difficult or too demanding. In this aspect, a disability pension may be used as a social security net to assist those who struggle with either their work or general life situation without meeting the formal requirements for a disability pension.

No previous studies have - to our knowledge - directly estimated what proportion of the relationship between SES and disability pension that can be explained by health. However, in one study from 2007 on the effect of mastering on the relationship between SES and disability pension, the authors adjusted for self-reported health [30]. With this adjustment the OR for disability pension was reduced from 5.7 to 4.1 for the subjects with the lowest level of education compared to those with the highest level [30]. These numbers are similar to those found in the present study. It should however be noted that in the 2007 study, health was only measured as a single dichotomous self-reported variable [30].

It is important to note that our study make claims solely about causes of *the difference *in prevalence of disability pension between groups of different level of education, and our findings must not be interpreted as to imply that health is not an important factor in explaining the overall prevalence of disability pension in any of these groups.

### Limitations

Our results must be viewed in light of four important limitations. First, in this population-based survey, the participation rate was 63%, which in turn was reduced to 51% after exclusions (Figure 1). Studies of non-participants in public health studies have shown that the probability of non-responding is greater for those with less education, older age, poorer health status, and for those on a disability pension [32-34]. On the other hand, population based health surveys like HUSK provide a large number of participants and give the possibility to adjust for a wide range of health measures. Finally they can be linked to comprehensive national registries of disability pensions like that of NIA. As such, population based studies are probably the best available option for the purpose of studying the association between SES, health and disability pension.

Secondly, this study was cross-sectional, with limited possibilities to address causality, and data on health were collected after initiation of disability pension (mean time 2.6 years). During the time from disability pension award to health assessment, some health problems might have diminished, and this would reduce the apparent effect of health on the connection between SES and disability pension. Also, psychological symptoms triggered by workplace problems would likely be relieved when on disability pension. However, some of our measures, like those of somatic diagnoses, are probably rather stable over time. Although the scientific documentation is ambiguous in this area, some studies indicate that health problems may actually increase after disability pension [35,36], whereas a previous study based on the HUSK data material indicated improvement in global health after a disability pension had been granted [37]. To the extent that a disability pension award increased or produced health problems we may have overestimated the proportion of the socioeconomic gradient in disability pension award that can be attributed to health. Finally, if health problems actually did diminish after disability pensioning, the result of our study would at least indicate that the *sustainment *of some disability pensions can not be justified on the argument of health.

Third, attendants were all 39-46 years old at participation, so it is uncertain if our results apply to other age groups. However, for studying the younger age groups, level of education might not be as good a measure of SES, as a disability pension granted early in life could influence on the level of education reached.

Fourth, it is possible that there are health problems leading to disability pension that were not covered by the HUSK questionnaire, or that the included health variables did not properly assess differences in intensity or severity of symptoms [38]. Even though our list of health variables was not complete, it covered a wide range of disorders and health problems. The collection of information about the regular use of medical substances, probably covered some diagnoses not directly assessed by the questionnaire. The tendency was for all of the measured variables to have little adjusting effect on the relationship between education and disability pension. We therefore doubt that any health problems we might have overlooked would have influenced the outcome notably. In the continuous variables (anxiety, depression and symptoms of somatisation), different levels of severity in the health problems were probably well reflected. However, for the dichotomous variables (fibromyalgia and localized pain), the intensity of the health problem should ideally have been measured, as there might have been socioeconomic gradients in the severity of these symptoms, and this could have underestimated the effect of health.

Moreover, as we wanted to examine what part of the relationship between low socioeconomic status and disability pension was not explained by health, we only adjusted for diseases that could be a direct cause of disability pension, deliberately leaving out factors such as smoking. While smoking is an important factor in most health issues, and might undeniably *cause *health problems, smoking in itself is neither a disease nor a reason for being granted a disability pension. The same reasoning led to the decision of leaving out blood pressure. Moderately hypertension is in itself not a disease, and would most likely not be considered a valid reason for disability pension. Cases of hypertension so severe as to be a reason for disability pension would most likely be treated with antihypertensive drugs, which again would be covered by our variable on number of pharmacological treatments.

## Conclusions

There is a socioeconomic gradient in disability pension corresponding to socioeconomic gradients in health. But the two gradients seem to be somewhat independent of each other; adjusting for health factors decreased the odds ratio for disability pension only slightly, and thus health seems to account for only a small part of the increased risk of disability pension in those with low SES. Further studies are needed to identify which factors other than health are of importance in explaining socioeconomic gradients in disability pension.

## List of abbreviations used

BMI: Body mass index; HADS: Hospital Anxiety and Depression Scale; HADS-A: Hospital Anxiety and Depression Scale - Anxiety subscale; HADS-D: Hospital Anxiety and Depression Scale - Depression subscale; HUSK: the Hordaland Health Study; NIA: (the Norwegian) National Insurance Administration; OECD: Organisation for Economic Co-operation and Development; OR: Odds ratio; ORs: Odds ratios; SES: Socioeconomic status;

## Competing interests

The authors declare that they have no competing interests.

## Authors' contributions

KAØ, REØ, TR and AM planned the study, KAØ and AKK performed the statistical analyses, and KAØ wrote the manuscript. All authors have taken part in the academic discussions of the manuscript's content and have contributed to revised versions of the manuscript. All authors have approved the final version.

## Pre-publication history

The pre-publication history for this paper can be accessed here:

http://www.biomedcentral.com/1471-2458/11/12/prepub

## References

[B1] Disability programmes in need of reform - a policy brief 2003 OECD Publishing

[B2] Sickness, Disability and Work: Breaking the Barriers (Vol. 1): Norway, Poland and Switzerland 2006 OECD Publishing

[B3] The Norwegian Department of Finance Stortingsmelding nr 9 2009

[B4] Adler NE Ostrove JM Socioeconomic status and health: What we know and what we don't Socioeconomic Status and Health in Industrial Nations - Social, Psychological, and Biological Pathways 1999 896 3 15 10.1111/j.1749-6632.1999.tb08101.x 10681884

[B5] Bratsberg B Fevang E Roed K Disablity in the welfare state: an unemployment problem in disguise? 2010 4897 IZA DP

[B6] Brun C Boggild H Eshoj P Socioeconomic risk indicators for disability pension within the Danish workforce. A registry-based cohort study of the period 1994-1998 Ugeskr Laeger 2003 165 3315 9 14531369

[B7] Krokstad S Johnsen R Westin S Social determinants of disability pension: a 10-year follow-up of 62 000 people in a Norwegian county population Int J Epidemiol 2002 31 1183 91 10.1093/ije/31.6.1183 12540720

[B8] Wikman A Marklund S Alexanderson K Illness, disease, and sickness absence: an empirical test of differences between concepts of ill health J Epidemiol Community Health 2005 59 450 4 10.1136/jech.2004.025346 15911638 PMC1757037

[B9] Brun C Boggild H Eshoj P Socioeconomic risk indicators for disability pension within the Danish workforce. A registry-based cohort study of the period 1994-1998 Ugeskr Laeger 2003 165 3315 9 14531369

[B10] Marmot M Inequalities in health New England Journal of Medicine 2001 345 134 6 10.1056/NEJM200107123450210 11450663

[B11] Allebeck P Mastekaasa A Swedish Council on Technology Assessment in Health Care (SBU). Chapter 5. Risk factors for sick leave - general studies Scand J Public Health Suppl 2004 63 49 108 10.1080/14034950410021853 15513654

[B12] Lynch JW Kaplan GA Salonen JT Why do poor people behave poorly? Variation in adult health behaviours and psychosocial characteristics by stages of the socioeconomic lifecourse Social Science & Medicine 1997 44 809 19 10.1016/s0277-9536(96)00191-8 9080564

[B13] Akselsen A Lien S Sandnes T FD-trygd dokumentasjonsrapport. Pensjoner. Grunn og hjelpestønader 1992-2001 2003 Oslo: Rikstrygdeverket

[B14] Chevalier A Luce D Blanc C Goldberg M Sickness absence at the French National Electric and Gas Company Br J Ind Med 1987 44 101 10 3814541 10.1136/oem.44.2.101 PMC1007790

[B15] Feeney A North F Head J Canner R Marmot M Socioeconomic and sex differentials in reason for sickness absence from the Whitehall II Study Occup Environ Med 1998 55 91 8 10.1136/oem.55.2.91 9614392 PMC1757555

[B16] Singh-Manoux A Clarke P Marmot M Multiple measures of socio-economic position and psychosocial health: proximal and distal measures International Journal of Epidemiology 2002 31 1192 9 10.1093/ije/31.6.1192 12540721

[B17] Winkleby MA Jatulis DE Frank E Fortmann SP Socioeconomic-Status and Health - How Education, Income, and Occupation Contribute to Risk-Factors for Cardiovascular-Disease American Journal of Public Health 1992 82 816 20 10.2105/AJPH.82.6.816 1585961 PMC1694190

[B18] Mykletun A Overland S Dahl AA Krokstad S Bjerkeset O Glozier N A population-based cohort study of the effect of common mental disorders on disability pension awards American Journal of Psychiatry 2006 163 1412 8 10.1176/appi.ajp.163.8.1412 16877655

[B19] Knudsen AK Overland S Aakvaag HF Harvey SB Hotopf M Mykletun A Common mental disorders and disability pension award: seven year follow-up of the HUSK study J Psychosom Res 2010 69 59 67 10.1016/j.jpsychores.2010.03.007 20630264

[B20] Zigmond AS Snaith RP The Hospital Anxiety and Depression Scale Acta psychiatr scand 1983 361 70 10.1111/j.1600-0447.1983.tb09716.x 6880820

[B21] Bjelland I Dahl AA Haug TT Neckelmann D The validity of the Hospital Anxiety and Depression Scale - An updated literature review Journal of Psychosomatic Research 2002 52 69 77 10.1016/S0022-3999(01)00296-3 11832252

[B22] Gjesdal S Kristiansen AM Norwegian fibromyalgia epidemic--its rise or possible decline. What is the trend based on disability statistics? Tidsskr Nor Laegeforen 1997 117 2449 53 9265303

[B23] Mykletun A Heradstveit O Eriksen K Glozier N Overland S Maeland JG Health anxiety and disability pension award: The HUSK Study Psychosom Med 2009 71 353 60 10.1097/PSY.0b013e31819cc772 19321853

[B24] Krokstad S Westin S Disability in society-medical and non-medical determinants for disability pension in a Norwegian total county population study Soc Sci Med 2004 58 1837 48 10.1016/S0277-9536(03)00409-X 15020001

[B25] Bruusgaard D Evensen AR Bjerkedal T Fibromyalgia - A New Cause for Disability Pension Scandinavian Journal of Social Medicine 1993 21 116 9 8367676 10.1177/140349489302100209

[B26] Harvey SB Glozier N Carlton O Mykletun A Henderson M Hotopf M Obesity and sickness absence: results from the CHAP study Occup Med (Lond) 2010 10.1093/occmed/kqq031 20308262

[B27] Kark M Neovius M Rasmussen F Obesity status and risk of disability pension due to psychiatric disorders International Journal of Obesity 2010 34 726 32 10.1038/ijo.2009.298 20101246

[B28] McKnight PE McKnight KM Sidani S Figueredo AJ Chapter 7: Data deletion methods for handling missing data Missing data - A gentle introduction 2007 The Guildford Press

[B29] McKnight PE McKnight KM Sidani S Figueredo AJ Chapter 9: Single imputation procedures Missing data - A gentle introduction 2007 The Guildford Press

[B30] Valset K Naper SO Claussen B Dalgard OS Does mastering have an effect on disability pensioning independent of health, and may it explain divides of education in the Oslo Health Survey? Scandinavian Journal of Public Health 2007 35 157 63 10.1080/14034940600984635 17454919

[B31] Henderson M Hotopf M Leon DA Childhood temperament and long-term sickness absence in adult life British Journal of Psychiatry 2009 194 220 3 10.1192/bjp.bp.107.044271 19252149

[B32] Korkeila K Suominen S Ahvenainen J Ojanlatva A Rautava P Helenius H Non-response and related factors in a nation-wide health survey European Journal of Epidemiology 2001 17 991 9 10.1023/A:1020016922473 12380710

[B33] Drivsholm T Eplov LF Davidsen M Jorgensen T Ibsen H Hollnagel H Representativeness in population-based studies: A detailed description of non-response in a Danish cohort study Scandinavian Journal of Public Health 2006 34 623 31 10.1080/14034940600607616 17132596

[B34] Knudsen AK Hotopf M Skogen JC Overland S Mykletun A The Health Status of Nonparticipants in a Population-based Health Study: The Hordaland Health Study Am J Epidemiol 2010 20843863 10.1093/aje/kwq257

[B35] Dave D Rashad I Spasojevic J The Effects of Retirement on Physical and Mental Health Outcomes Southern Economic Journal 2008 75 497 523

[B36] Tuomi K Jarvinen E Eskelinen L Ilmarinen J Klockars M Effect of Retirement on Health and Work Ability Among Municipal Employees Scandinavian Journal of Work Environment & Health 1991 17 75 81 1792534

[B37] Overland S Glozier N Henderson M Maeland JG Hotopf M Mykletun A Health status before, during and after disability pension award: the Hordaland Health Study (HUSK) Occup Environ Med 2008 65 769 73 10.1136/oem.2007.037861 18940958

[B38] Overland S Glozier N Maeland JG Aaro LE Mykletun A Employment status and perceived health in the Hordaland Health Study (HUSK) BMC Public Health 2006 6 10.1186/1471-2458-6-219 16939642 PMC1560129

